# Audience attitudes and aesthetic perception of digitally empowered contemporary lacquer painting: a survey-based technology-imagery-perception model

**DOI:** 10.3389/fpsyg.2026.1866076

**Published:** 2026-09-03

**Authors:** Yuanyuan Liu

**Affiliations:** School of Art, Huangshan University, Tunxi, Anhui, China

**Keywords:** aesthetic perception, digital empowerment, lacquer painting imagery characteristics, technology acceptance model, virtual-real integration

## Abstract

Contemporary lacquer painting faces challenges of audience engagement, interpretive accessibility, and digital dissemination. To avoid conflating actual technology exposure with respondents’ beliefs about technology, this study is positioned as a survey-based examination of audience attitudes toward digitally empowered lacquer painting. A technology-imagery-perception (TIP) model is used to examine whether perceived immersive, interactive, and distributed affordances are associated with three imagery characteristics: sensible scene, imaginable atmosphere, and virtual-real imagination. Data from 425 valid questionnaires were analyzed using reliability and validity tests, correlations, multiple regression, an interaction analysis, and a constrained bootstrap serial indirect-association analysis. Perceived immersive, interactive, and distributed affordances were positively associated with sensible scene, imaginable atmosphere, and virtual-real imagination, respectively. The three imagery characteristics were also positively associated with emotional resonance, cognitive evaluation, and behavioral intention. The constrained model identified two non-causal indirect components through emotional resonance alone and through emotional resonance followed by cognitive evaluation; technology acceptance also conditioned the virtual-real-imagination–behavioral-intention association. Because the evidence is cross-sectional and self-reported, these findings describe associations among perceptions and attitudes rather than causal effects of actual digital technologies.

## 1 Introduction

Traditional lacquer painting is an important carrier of Eastern cultural heritage, and its material luster, layered craftsmanship, and symbolic imagery constitute distinctive aesthetic value (Kim, 2014). In the digital environment, however, lacquer painting also faces problems of limited audience contact, weak interpretive accessibility, and fragmented online dissemination (Song et al., 2019). The present study therefore focuses on a specific research object: audience aesthetic perception of digitally empowered contemporary lacquer painting. Rather than evaluating the technical performance of VR, AR, blockchain, or NFT systems, the study examines how audiences’ perceived technological affordances are associated with their psychological perception of lacquer painting imagery and their subsequent aesthetic responses.

The research gap lies in the insufficient explanation of the psychological mechanism linking digital technology discourse and audience aesthetic perception in traditional art communication (Hu and Foo, 2025). Existing studies on digital heritage and lacquer art often discuss restoration, exhibition design, or dissemination platforms, while media psychology studies emphasize emotional resonance, cognitive evaluation, and behavioral intention. Few studies connect these two strands through measurable imagery characteristics (Manovich, 2001; Bolter and Grusin, 1999; Liang, 2025). Accordingly, this study treats immersive, interactive, and distributed technologies as perceived affordances reported by respondents, and examines whether these affordances are associated with sensible scene, imaginable atmosphere, and virtual-real imagination.

From the perspective of media psychology, audience aesthetic perception can be understood as a process in which sensory cues, imagery interpretation, emotional resonance, cognitive evaluation, and behavioral intention are interrelated. Classical pragmatist aesthetics treats art reception as an experiential process (Dewey, 1934), while neuroaesthetic and embodied-cognition studies provide conceptual context for the emotional and cognitive dimensions of art reception (Zeki et al., 2020; Coëgnarts, 2025). The present study does not report physiological sensing or eye-tracking indicators (Skryabin, 2026); its empirical evidence is limited to cross-sectional self-report data. Emotional resonance and cognitive evaluation are therefore operationalized as questionnaire constructs, and the results are interpreted as statistical associations among perceived technological affordances, imagery characteristics, and audience responses (Welke and Vessel, 2025; Chen et al., 2025; Mikalonytė et al., 2026; Cheron and De Maere, 2025).

Accordingly, this study examines the survey-based relationships linking perceived digital technology affordances, lacquer-painting imagery characteristics, and audiences’ aesthetic perceptions. The research questions are threefold: how perceived immersive, interactive, and distributed affordances are associated with the perception of lacquer-painting imagery; how imagery characteristics are associated with emotional resonance, cognitive evaluation, and behavioral intention; and whether the observed variables form a statistically significant sequential indirect-association pattern. Questionnaire-based measurement, reliability and validity testing, regression, interaction analysis, and bootstrap indirect-path analysis are used to evaluate the proposed model.

## 2 Construction of the theoretical model

### 2.1 Definition of core concepts

Digital empowerment technology in this paper refers to perceived technological affordances rather than directly manipulated technical conditions. Specifically, immersive affordance refers to respondents’ belief that a digital system could provide controllable viewpoints, zoom functions, and mediated viewing presence (Dağ et al., 2026; Rauschnabel et al., 2019); interactive affordance refers to respondents’ belief that a digital system could support selectable information paths, comparison, and audience contribution (Nikolaou, 2024; Burke, 2020; Chen and Young, 2026); distributed affordance refers to respondents’ belief that digital infrastructure could provide traceable provenance, licensing records, and authorized network access (Valeonti et al., 2021; Nadini et al., 2021). These definitions keep the technology layer focused on system capabilities and separate it from perceived qualities of the artwork.

The imagery characteristics of lacquer painting are defined as audience-perceived aesthetic qualities generated through the interaction of material texture, symbolic form, and display context (Xiao et al., 2024; Balderrama-Armendariz et al., 2025). Sensible scene emphasizes perceived material tangibility, layered surfaces, luster, visual depth, and spatial concreteness. Imaginable atmosphere emphasizes narrative association, cultural context, and situational meaning generated by the work. Virtual-real imagination emphasizes conceptual continuity between the physical artwork and its digital representation, rather than the technical functions of distribution platforms. These three constructs are measured as artwork-referent imagery perceptions.

### 2.2 Construction of theoretical model

The technology-imagery-perception model is a psychological perception model rather than a design-performance model. The technology layer includes three perceived affordances: immersive, interactive, and distributed affordance. The imagery layer includes three audience-perceived characteristics: sensible scene, imaginable atmosphere, and virtual-real imagination. The response layer includes emotional resonance, cognitive evaluation, and behavioral intention. Technology acceptance is examined as an attitudinal moderator (Dieste et al., 2025; Lin et al., 2025). This configuration makes explicit that the study concerns respondents’ subjective perceptions and expectations, not the objective performance or causal outcomes of actual VR, AR, NFT, or blockchain interventions.

The proposed associations between technology affordances and imagery characteristics are theoretically differentiated. Perceived immersive affordance should be positively associated with sensible scene because it concerns the viewing capabilities that respondents believe digital media could provide. Perceived interactive affordance should be positively associated with imaginable atmosphere because selectable exploration and participatory interpretation may correspond to stronger narrative association. Perceived distributed affordance should be positively associated with virtual-real imagination because authentication, circulation, and derivative participation concern the perceived connection between physical works and digital extensions. The final questionnaire wording in Appendix A maintains these distinct referents.

The proposed relationships from imagery characteristics to aesthetic-response indicators follow a progressive interpretive logic. Sensible scene concerns the perceived material and spatial concreteness of the work and is expected to be associated with emotional resonance. Imaginable atmosphere concerns narrative and cultural association and is expected to be associated with cognitive evaluation (Du et al., 2025). Virtual-real imagination concerns perceived continuity between physical and digital forms and is expected to be associated with willingness to share, revisit, recommend, or participate (Yan and Du, 2025). These constructs may therefore form an ordered statistical pattern across emotion, cognition, and behavioral intention (Jiang et al., 2023; Qaisar et al., 2024; Tan, 2024; Yang et al., 2024), although temporal order cannot be established with cross-sectional data.

To reduce construct overlap, the formal questionnaire applied a strict item-level referent rule and a wording audit before data collection. Technology-affordance items describe an action or capability attributable to a digital system, such as zooming, switching viewpoints, selecting information paths, recording provenance, or controlling authorized access. Imagery-characteristic items describe qualities attributed to the artwork, such as material tangibility, narrative atmosphere, or conceptual continuity between physical and digital versions. Aesthetic-response items describe the respondent’s affective, cognitive, or behavioral-intention state. Potentially overlapping pairs were rewritten: for example, the immersive-affordance item refers to zooming and viewpoint control, whereas the sensible-scene item refers to perceived luster and layered material quality; the distributed-affordance item refers to provenance or licensing records, whereas the virtual-real-imagination item refers to the perceived unity of physical and digital versions. Appendix A reports the final wording used in the formal survey.

### 2.3 Proposal of research hypotheses

Based on immersive-media theory, perceived immersive affordance is expected to be positively associated with audiences’ sense that digital media could provide controllable viewing access. Hypothesis H1 is proposed: perceived immersive affordance is positively associated with sensible scene.

Based on interaction-design theory, perceived interactive affordance is expected to be positively associated with participation in interpretation and narrative exploration. Hypothesis H2 is proposed: perceived interactive affordance is positively associated with imaginable atmosphere.

Based on distributed-media theory, perceived distributed affordance is expected to be positively associated with respondents’ imagination of authentication, circulation, derivative participation, and the physical-digital boundary of lacquer painting. Hypothesis H3 is proposed: perceived distributed affordance is positively associated with virtual-real imagination.

Research on aesthetic perception suggests that perceived material and spatial concreteness should be positively associated with emotional resonance. Hypothesis H4 is proposed: sensible scene is positively associated with emotional resonance. Narrative and cultural association should be positively related to interpretive understanding. Hypothesis H5 is proposed: imaginable atmosphere is positively associated with cognitive evaluation. Perceived continuity between physical and digital forms should be positively related to sharing and participation intentions. Hypothesis H6 is proposed: virtual-real imagination is positively associated with behavioral intention. Finally, the variables may form an ordered indirect statistical pattern. Hypothesis H7 is proposed: sensible scene is positively associated with behavioral intention through the prespecified serial sequence of emotional resonance and cognitive evaluation. The shorter sensible scene → emotional resonance → behavioral intention component is also reported to provide a complete decomposition of the constrained model.

## 3 Research design

### 3.1 Questionnaire design

**Figure 1:**
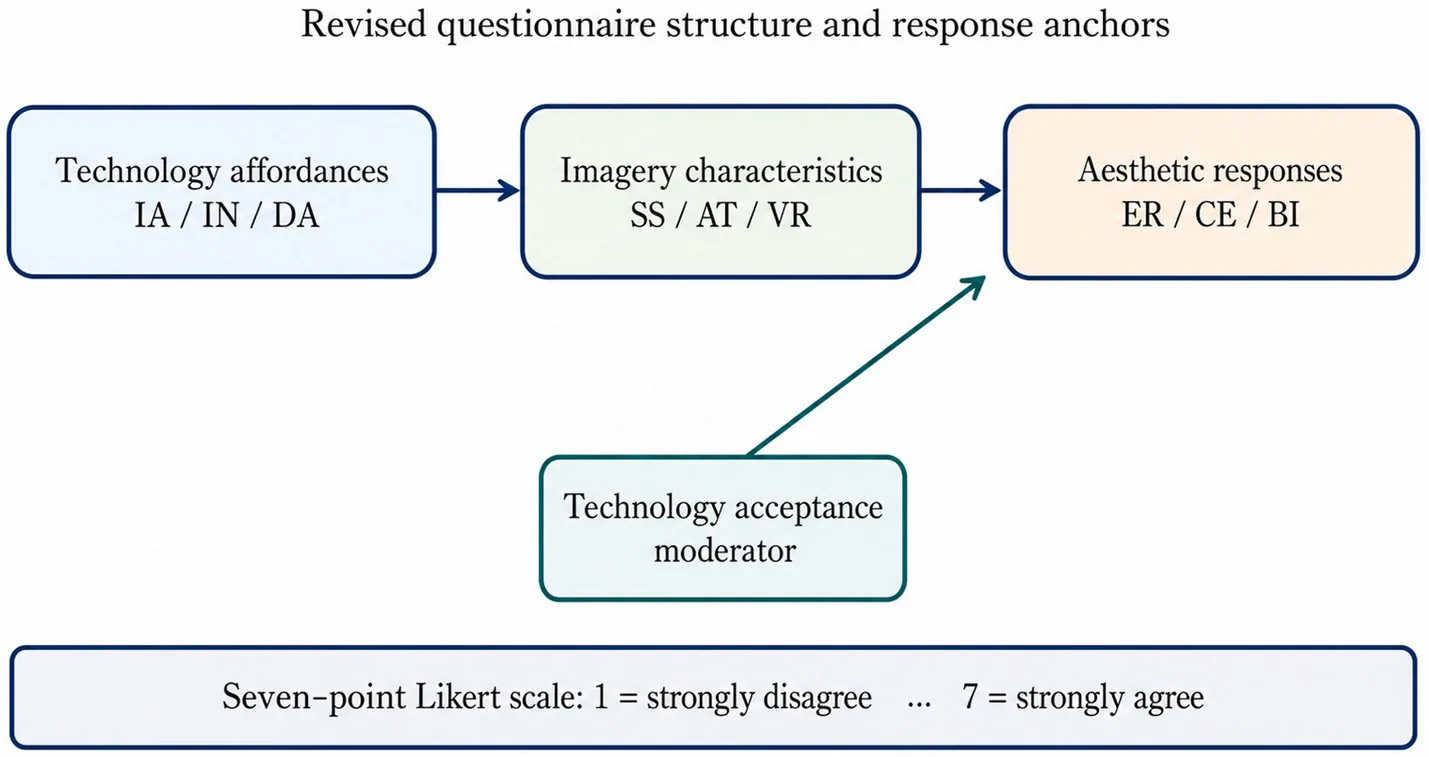
Survey measurement domains and standardized seven-point response anchors.

The questionnaire uses a standardized seven-point Likert scale for all substantive items, with 1 = strongly disagree and 7 = strongly agree. Figure 1 presents the survey measurement domains and response anchors rather than an experimental procedure. The formal scale contains 10 latent variables and 30 items, with three items for each construct. Technology variables measure perceived system capabilities rather than actual exposure. Examples include: “A digital interface could let me zoom, rotate, or switch viewpoints when examining a lacquer painting”; “A digital platform could enable viewers to contribute interpretations or responses”; and “Digital authentication tools could provide traceable provenance records.” Appendix A reports the complete final item set.

The lacquer-painting imagery characteristics were measured with three artwork-referent constructs. Sensible-scene items concern material tangibility, layered surfaces, luster, and visual depth. Imaginable-atmosphere items concern narrative setting, cultural association, and situational meaning. Virtual-real-imagination items concern conceptual continuity between the physical work, its digital representation, and circulation across material and digital forms. These items do not ask whether a technology performs a function.

Aesthetic response was measured through emotional resonance, cognitive evaluation, and behavioral intention. Emotional-resonance items capture affective engagement; cognitive-evaluation items capture cultural understanding and interpretive clarity; behavioral-intention items capture willingness to share, revisit, recommend, or participate in related activities. Technology acceptance is measured separately through familiarity, perceived usefulness, and perceived ease of use. The separation of system capability, artwork quality, and respondent response reduces semantic redundancy across the measurement domains.

### 3.2 Sample design, recruitment, and data collection

**Figure 2:**
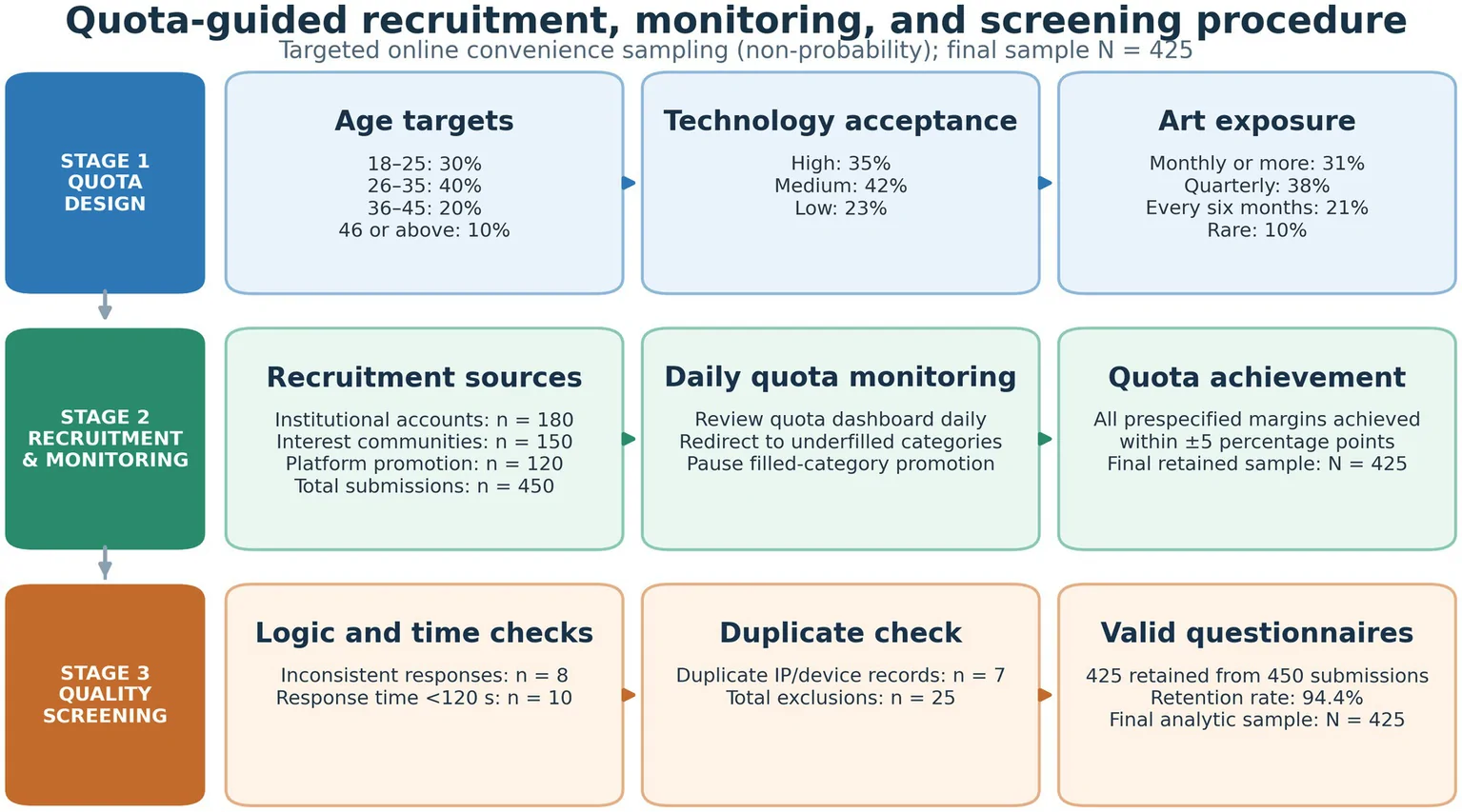
Quota-guided targeted online recruitment, marginal-quota monitoring, and screening procedure.

The study used quota-guided targeted online convenience sampling, not probability-based stratified sampling. Before recruitment, marginal composition targets were set for age (30% aged 18–25, 40% aged 26–35, 20% aged 36–45, and 10% aged 46 or above), technology acceptance (35% high, 42% medium, and 23% low), and art-exposure frequency (31% monthly or more, 38% quarterly, 21% every 6 months, and 10% rarely). These targets were intended to preserve analytical heterogeneity across groups rather than reproduce known population parameters. Because participation was voluntary and no sampling frame or random selection was used, the resulting sample should not be described as statistically representative (see Figure 2).

The *a priori* power calculation was rechecked in G*Power 3.1 using the linear multiple-regression option for a fixed model and deviation of *R*^2^ from zero. The most parameterized hierarchical regression contained five simultaneous predictors: virtual-real imagination, technology acceptance, their mean-centered interaction term, gender, and education level. With *f*^2^ = 0.15, *α* = 0.05, power = 0.95, and five predictors, the minimum required sample was 138. A substantially more conservative target of at least 400 valid cases was adopted to support the 10-factor measurement model, 5,000-sample bootstrap estimates, and technology-acceptance subgroup descriptions. Allowing for invalid online responses, 450 questionnaires were collected; 425 were retained after screening.

Recruitment was conducted through Wenjuanxing using three targeted channels: art-college and art-museum public accounts, digital-art and lacquer-art interest communities, and the platform’s targeted promotion function. The research team reviewed the marginal quota dashboard each day. Invitations were redirected toward under-filled age, technology-acceptance, or art-exposure categories, and promotion to a filled category was paused when necessary. The quotas were monitored marginally rather than as a fully cross-classified sampling matrix. Returns comprised 180 questionnaires from institutional public accounts, 150 from interest communities, and 120 from targeted promotion. This procedure improved compositional coverage, but it did not remove self-selection or channel-selection bias.

**Figure 3:**
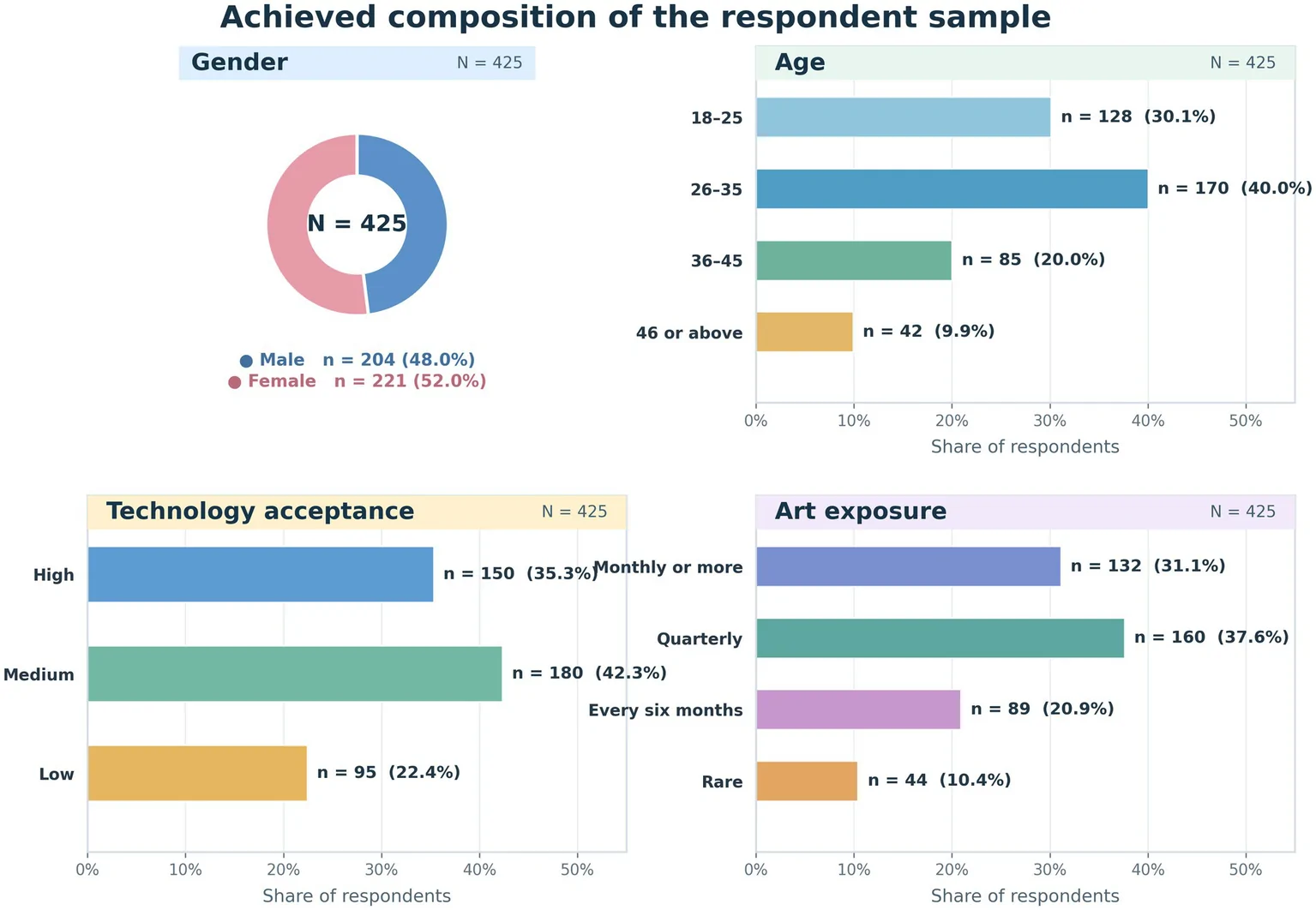
Achieved composition of the 425 valid respondents; percentages describe the sample rather than population parameters.

Among the 425 valid cases, 204 respondents were male (48.0%) and 221 were female (52.0%). The age distribution was 128 aged 18–25 (30.1%), 170 aged 26–35 (40.0%), 85 aged 36–45 (20.0%), and 42 aged 46 or above (9.9%). Eighty respondents (18.8%) had a college education or below, and 345 (81.2%) had a bachelor’s degree or above. Technology acceptance was high for 150 respondents (35.3%), medium for 180 (42.3%), and low for 95 (22.4%). Art exposure was monthly or more for 132 respondents (31.1%), quarterly for 160 (37.6%), every 6 months for 89 (20.9%), and rare for 44 (10.4%). Figure 3 describes the achieved sample composition; it should not be read as evidence that these proportions represent the wider public.

Data cleaning followed three pre-specified criteria. Eight questionnaires with logically contradictory answers were removed, 10 completed in less than 120 s were removed, and seven duplicate submissions identified through platform IP/device information were removed. Thus, 25 cases were excluded and 425 valid cases remained (valid-response rate = 94.4%). For the core variables, absolute skewness was below 1 (range: −0.78 to 0.82) and absolute kurtosis was below 2 (range: −1.15 to 1.28). Harman’s single-factor test yielded 27.6% variance for the first factor. This diagnostic does not eliminate common-method bias, but it indicates that a single factor did not dominate the covariance structure.

### 3.3 Pre-testing, reliability and validity testing

A pre-test questionnaire was distributed through Wenjuanxing to art students, digital-art enthusiasts, and followers of intangible cultural heritage. Of 150 returns, 138 were valid (92.0%). The pre-test sample was used for item clarity checks, preliminary reliability analysis, and exploratory factor analysis; it was not pooled with the formal sample. Ambiguous or semantically overlapping wording was revised before formal distribution. The final questionnaire retained 30 items for 10 latent variables, with three items per construct. The independent formal sample of 425 respondents was then used for descriptive analysis, correlations, regression, bootstrap path analysis, confirmatory factor analysis, and discriminant-validity testing.

**Figure 4:**
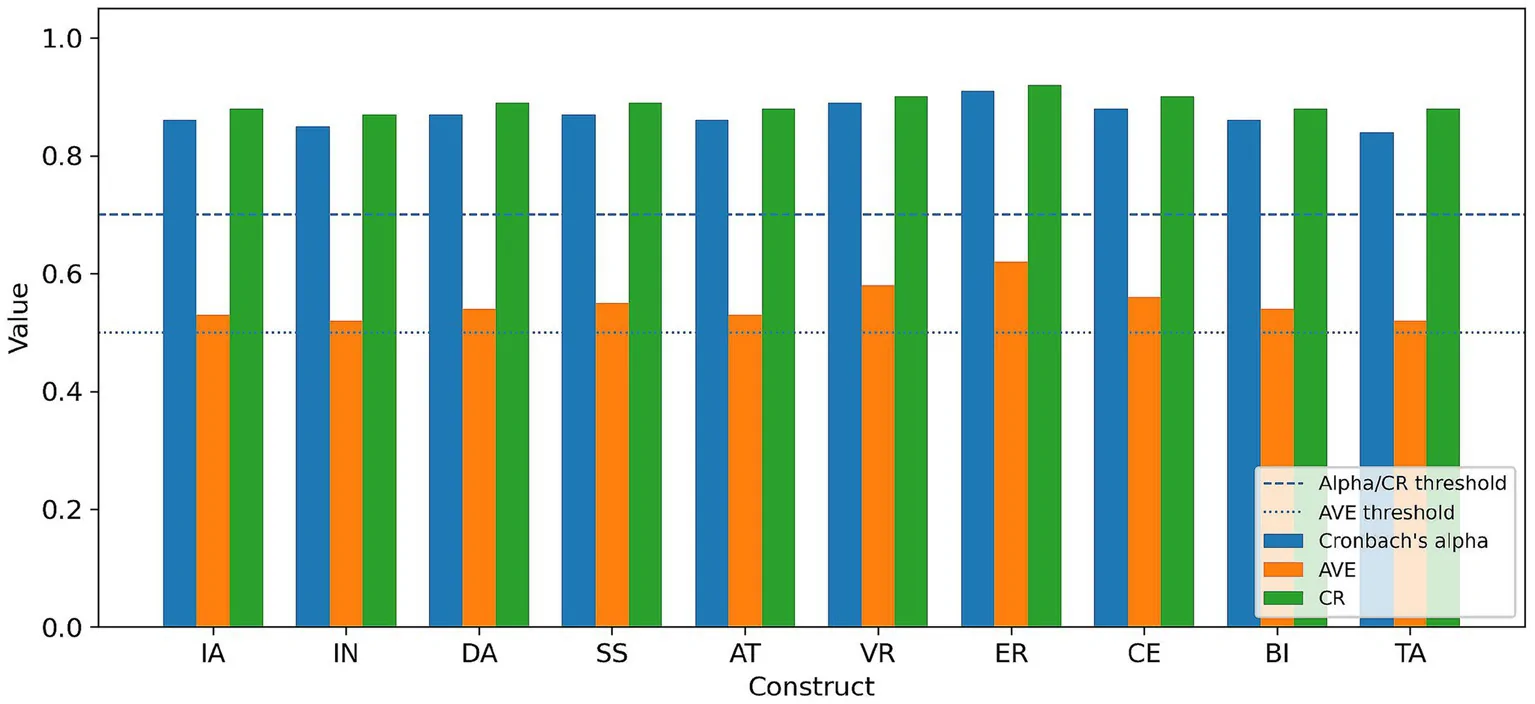
Reliability and convergent-validity indicators.

Pre-test reliability was assessed using Cronbach’s alpha, average variance extracted (AVE), and composite reliability (CR). Table 1 reports the results for the 138 valid pre-test cases. All alpha coefficients exceeded 0.84, all AVE values exceeded 0.52, and all CR values exceeded 0.87, indicating acceptable preliminary internal consistency and convergent validity. Figure 4 visualizes selected indicators. These statistics evaluate the questionnaire constructs; they do not imply that actual VR/AR, blockchain, or NFT systems were manipulated.

**Table 1:** Pre-test reliability and convergent-validity summary (*N* = 138).

Latent variable	Items retained	Cronbach’s *α*	AVE	CR
Immersive affordance	3	0.86	0.53	0.88
Interactive affordance	3	0.85	0.52	0.87
Distributed affordance	3	0.87	0.54	0.89
Sensible scene	3	0.87	0.55	0.89
Imaginable atmosphere	3	0.86	0.53	0.88
Virtual-real imagination	3	0.89	0.58	0.90
Emotional resonance	3	0.91	0.62	0.92
Cognitive evaluation	3	0.88	0.56	0.90
Behavioral intention	3	0.86	0.54	0.88
Technology acceptance	3	0.84	0.52	0.88

**Figure 5:**
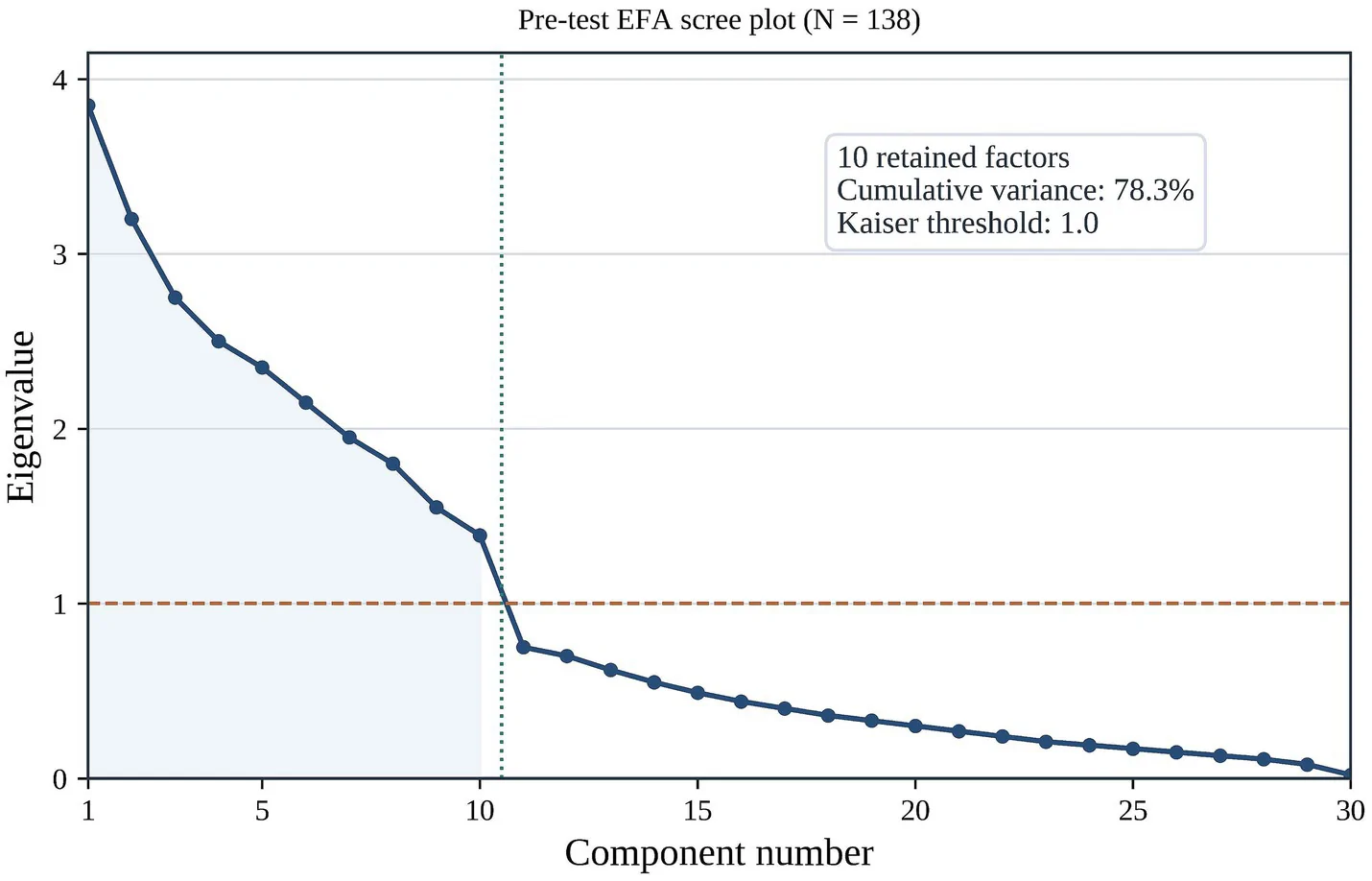
Full 30-component scree plot from the pre-test exploratory factor analysis (*N* = 138).

Exploratory factor analysis was conducted only on the 138-case pre-test sample using principal-axis factoring with Varimax rotation. The KMO value was 0.89, and Bartlett’s test of sphericity was significant (*χ*^2^ = 2136.72, *p* < 0.001), supporting factorability. The full 30-component scree plot is shown in Figure 5: the first 10 eigenvalues exceeded 1.0, the remaining 20 were below 1.0, and the 10 retained factors explained 78.3% of cumulative variance. Primary loadings ranged from 0.74 to 0.86, while the maximum cross-loading was below 0.26 (Tables 2, 3; Appendix C, Table C1).

**Table 2:** Pre-test exploratory factor-analysis quality summary (*N* = 138).

Construct	Item code	Primary loading range	Maximum cross-loading	Pre-test decision
Immersive affordance	IA1-IA3	0.75–0.83	0.21	Retained
Interactive affordance	IN1-IN3	0.74–0.82	0.23	Retained
Distributed affordance	DA1-DA3	0.76–0.84	0.22	Retained
Sensible scene	SS1-SS3	0.77–0.85	0.20	Retained
Imaginable atmosphere	AT1-AT3	0.74–0.82	0.24	Retained
Virtual-real imagination	VR1-VR3	0.78–0.86	0.21	Retained
Emotional resonance	ER1-ER3	0.80–0.86	0.19	Retained
Cognitive evaluation	CE1-CE3	0.76–0.84	0.22	Retained
Behavioral intention	BI1-BI3	0.75–0.83	0.23	Retained
Technology acceptance	TA1-TA3	0.74–0.81	0.24	Retained

**Table 3:** Pre-test rotated factor solution (principal-axis factoring with Varimax rotation; *N* = 138).

Construct	Rotated factor	Rotated loading range	Variance explained (%)	Interpretation
Immersive affordance	F1	0.78–0.86	8.3	Perceived sensory affordance
Interactive affordance	F2	0.77–0.84	8.0	Perceived participatory affordance
Distributed affordance	F3	0.79–0.85	7.8	Perceived circulation affordance
Sensible scene	F4	0.78–0.86	8.2	Material and spatial presence
Imaginable atmosphere	F5	0.76–0.83	7.6	Narrative and cultural imagination
Virtual-real imagination	F6	0.80–0.86	8.0	Physical-digital boundary perception
Emotional resonance	F7	0.81–0.87	8.1	Affective aesthetic response
Cognitive evaluation	F8	0.77–0.84	7.5	Interpretive understanding
Behavioral intention	F9	0.76–0.83	7.4	Sharing and participation intention
Technology acceptance	F10	0.75–0.82	7.4	Familiarity and perceived usefulness

Principal-axis factoring with Varimax rotation retained 10 first-order factors. The first 10 eigenvalues exceeded 1.0, the remaining 20 were below 1.0, and cumulative variance explained was 78.3%. KMO = 0.89; Bartlett’s test *p* < 0.001. Full item wording is provided in Appendix A.

Confirmatory and discriminant-validity analyses were then conducted on the independent formal sample (*N* = 425) using maximum-likelihood estimation. The 10-factor measurement model showed acceptable fit (*χ*^2^/df = 2.45, CFI = 0.94, TLI = 0.93, RMSEA = 0.06, and SRMR = 0.05; Appendix C, Table C2). Fornell-Larcker comparisons showed that the square root of AVE for each construct exceeded its correlations with other constructs, and all heterotrait-monotrait ratios were below 0.85 (Appendix C, Table C3). Using separate pre-test and formal samples avoids using the same observations for both exploratory item retention and confirmatory model evaluation. These checks reduce, but cannot eliminate, concerns about same-source construct overlap.

## 4 Empirical analysis

### 4.1 Descriptive statistical analysis

Descriptive statistics summarize the achieved online sample and the distributions of the core variables. Of the 425 valid respondents, 204 were male (48.0%) and 221 were female (52.0%); 70.1% were aged 18–35; 81.2% held a bachelor’s degree or above; technology acceptance was high, medium, and low for 35.3, 42.3, and 22.4%, respectively; and 31.1% reported art exposure at least monthly. These characteristics indicate that the sample was comparatively educated and engaged with art or digital media, a composition that is considered when interpreting external validity.

The descriptive statistical results of the core variables are presented in Table 4. The mean values of all core variables fall within the upper-middle range of the seven-point scale (3.95–4.35), indicating generally positive audience attitudes toward digitally empowered lacquer painting. Among them, the mean value of sensible scene is the highest (M = 4.35, SD = 0.98), suggesting that respondents most strongly perceived material texture, gloss, and spatial concreteness. The mean value of technology acceptance is 4.30 (SD = 1.09), indicating relatively high familiarity with digital art technologies. The mean value of perceived distributed affordance is comparatively lower (M = 3.95, SD = 1.23), which may reflect the lower public familiarity with distributed authentication and digital derivative mechanisms. The standard deviations range from 0.92 to 1.23, showing moderate dispersion across respondents.

**Table 4:** Descriptive statistical results of core variables (*N* = 425).

Variable name	Mean (M)	Standard deviation (SD)	Minimum value (Min)	Maximum (Max)
Perceived immersive affordance	4.12	1.05	1	7
Perceived interactive affordance	4.08	1.12	1	7
Perceived distributed affordance	3.95	1.23	1	7
Sensible scene	4.35	0.98	1	7
Imaginable atmosphere	4.22	1.01	1	7
Virtual-real imagination	4.05	1.15	1	7
Emotional resonance	4.28	0.92	1	7
Cognitive evaluation	4.15	1.03	1	7
Behavioral intention	4.02	1.18	1	7
Technology acceptance	4.30	1.09	1	7

The table data is based on a seven-point Likert scale (1 = completely disagree, 7 = completely agree). The mean reflects the overall perception level of the sample towards the variable, while the standard deviation reflects the degree of data dispersion.

The descriptive results are not used to evaluate the directional hypotheses. They show only that the sample reported generally positive mean attitudes and sufficient variation for subsequent association analyses. Sensible scene had the highest mean, whereas perceived distributed affordance had the lowest. Differences in technology acceptance across descriptive subgroups motivated the planned interaction and subgroup analyses but do not themselves establish moderation.

### 4.2 Correlation analysis

Pearson correlation analysis was used to examine the linear associations among core variables and to provide preliminary evidence for the regression models. KMO and Bartlett’s test are not used as evidence for correlation analysis; they are reported only in the factor-analysis section. The correlation analysis here focuses on coefficient size, significance level, and theoretical direction among technology affordances, imagery characteristics, aesthetic perception variables, and technology acceptance.

The Pearson correlation matrix of the core variables is presented in Table 5. Perceived immersive affordance is significantly correlated with sensible scene (*r* = 0.58, *p* < 0.01), perceived interactive affordance with imaginable atmosphere (*r* = 0.55, *p* < 0.01), and perceived distributed affordance with virtual-real imagination (*r* = 0.52, *p* < 0.01). These results preliminarily support the expected technology-affordance-to-imagery associations. The correlations between imagery characteristics and aesthetic perception are also significant: sensible scene and emotional resonance (*r* = 0.62, *p* < 0.01), imaginable atmosphere and cognitive evaluation (*r* = 0.59, *p* < 0.01), and virtual-real imagination and behavioral intention (*r* = 0.56, *p* < 0.01).

**Table 5:** Pearson correlation matrix of core variables (*N* = 425).

Variable name	1	2	3	4	5	6	7	8	9	10
1. Perceived immersive affordance	1	0.45**	0.38**	0.58**	0.32**	0.29**	0.41**	0.35**	0.33**	0.45**
2. Perceived interactive affordance		1	0.42**	0.31**	0.55**	0.35**	0.39**	0.42**	0.38**	0.43**
3. Perceived distributed affordance			1	0.28**	0.33**	0.52**	0.32**	0.36**	0.41**	0.42**
4. Sensible scene				1	0.48**	0.42**	0.62**	0.45**	0.43**	0.48**
5. Imaginable atmosphere					1	0.46**	0.51**	0.59**	0.47**	0.46**
6. Virtual-real imagination						1	0.43**	0.44**	0.56**	0.51**
7. Emotional resonance							1	0.58**	0.52**	0.44**
8. Cognitive evaluation								1	0.55**	0.43**
9. Behavioral intention									1	0.47**
10. Technology acceptance										1

**p < 0.01, *p < 0.05; diagonal indicates variable self-correlation.

**Figure 6:**
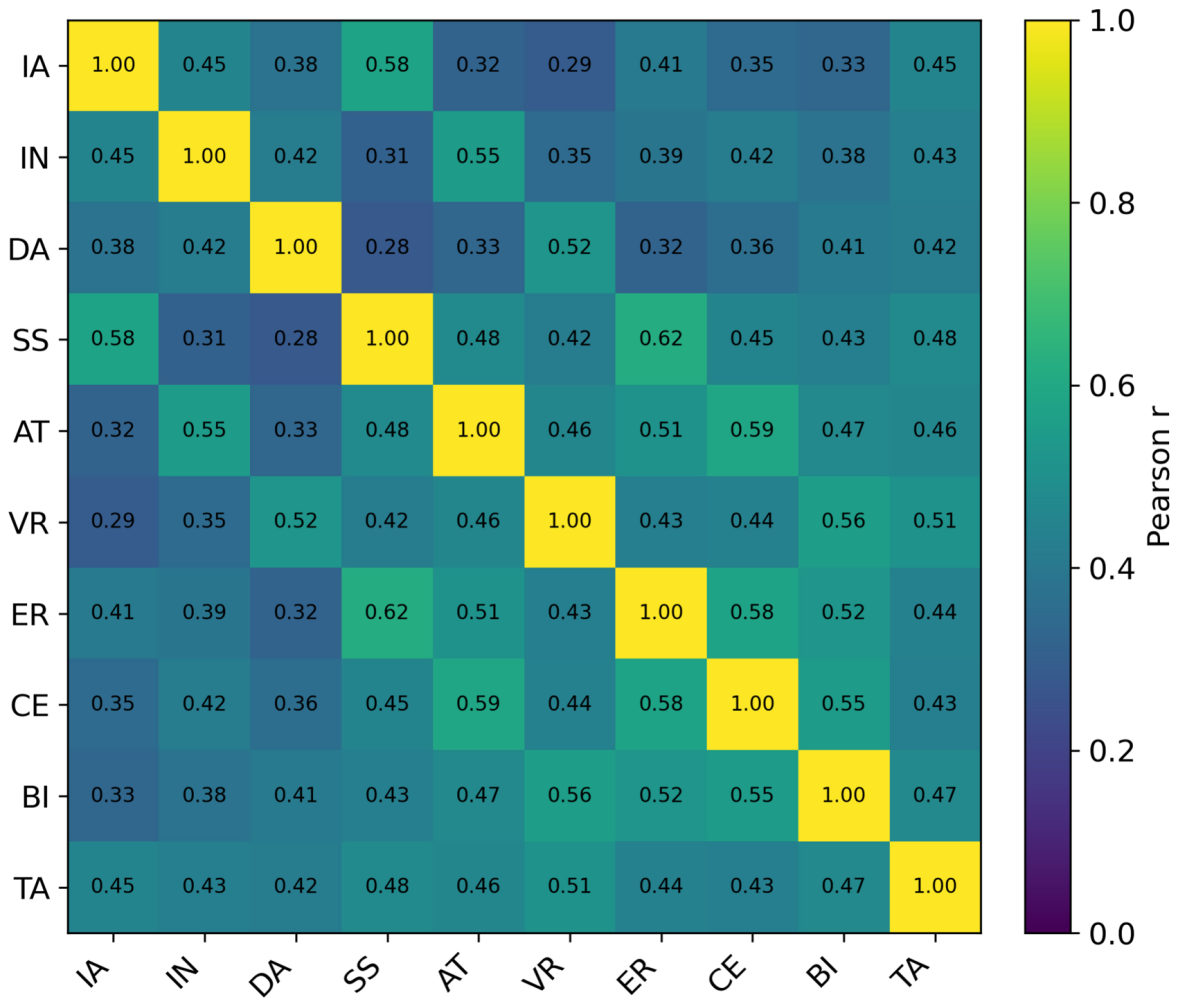
Correlation matrix of the core constructs.

Technology acceptance was positively correlated with all core variables (*r* = 0.42–0.51, *p* < 0.01), as shown in Figure 6. Its correlation with virtual-real imagination was the largest among these correlations (*r* = 0.51). This pattern justified testing a conditional association between virtual-real imagination and behavioral intention. Correlations involving the control variables were smaller, but their inclusion does not rule out residual demographic or channel-related confounding.

### 4.3 Multiple regression analysis

**Figure 7:**
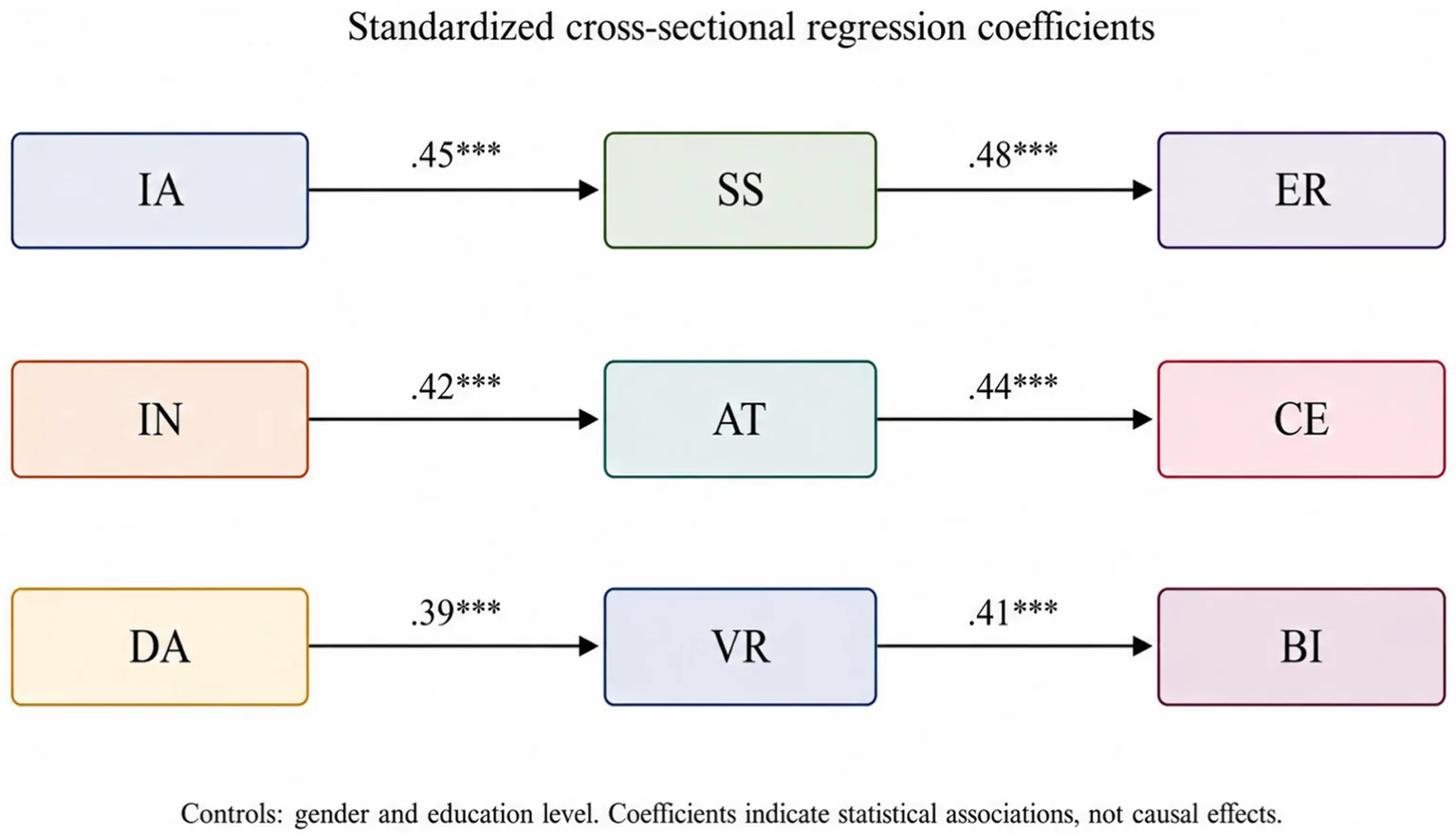
Standardized cross-sectional regression associations among the focal constructs.

Multiple regression used the forced-entry method and included gender (0 = male, 1 = female) and education level (1 = college or below, 2 = bachelor’s degree, 3 = master’s degree or above) as controls. Separate models examined associations between perceived technology affordances and imagery characteristics and between imagery characteristics and aesthetic-response indicators. Coefficients, standard errors, model fit, and significance tests were obtained in SPSS 27.0. Because the data are cross-sectional, the regression coefficients are interpreted as adjusted associations rather than causal path effects (see Figure 7).

Table 6 reports the adjusted associations between perceived technology affordances and imagery characteristics. All three models were statistically significant (*p* < 0.001). Perceived immersive affordance was positively associated with sensible scene (*β* = 0.45, *t* = 8.23, *p* < 0.001; adjusted *R*^2^ = 0.31), perceived interactive affordance with imaginable atmosphere (*β* = 0.42, *t* = 7.65, *p* < 0.001; adjusted *R*^2^ = 0.29), and perceived distributed affordance with virtual-real imagination (*β* = 0.39, *t* = 6.98, *p* < 0.001; adjusted *R*^2^ = 0.27). These results are consistent with H1-H3. The control coefficients were not significant in these models, but this does not establish demographic invariance.

**Table 6:** Multiple regression results for perceived affordances and imagery characteristics (*N* = 425).

Model	Dependent variable	Key predictor	B	SE	Beta	*t*	*p*	*R* ^2^	Adj. *R*^2^	Conclusion
1	Sensible scene	Perceived immersive affordance	0.43	0.052	0.45	8.23	<0.001	0.32	0.31	H1 supported
2	Imaginable atmosphere	Perceived interactive affordance	0.41	0.054	0.42	7.65	<0.001	0.30	0.29	H2 supported
3	Virtual-real imagination	Perceived distributed affordance	0.38	0.054	0.39	6.98	<0.001	0.28	0.27	H3 supported
Controls	All models	Gender; education level	-	-	n.s.	-	>0.05	-	-	Controlled

B, unstandardized coefficient; SE, standard error; *β*, standardized coefficient; *p*, significance probability. Gender and education level were entered simultaneously as control variables.

Table 7 reports the adjusted associations between imagery characteristics and aesthetic-response indicators. Sensible scene was positively associated with emotional resonance (*β* = 0.48, *t* = 9.12, *p* < 0.001; adjusted *R*^2^ = 0.34), imaginable atmosphere with cognitive evaluation (*β* = 0.44, *t* = 8.56, *p* < 0.001; adjusted *R*^2^ = 0.32), and virtual-real imagination with behavioral intention (*β* = 0.41, *t* = 7.89, *p* < 0.001; adjusted *R*^2^ = 0.30). These coefficients are consistent with H4-H6 and describe an ordered pattern of associations; they do not demonstrate temporal progression.

**Table 7:** Multiple regression results for imagery characteristics and aesthetic perception (*N* = 425).

Model	Dependent variable	Key predictor	B	SE	Beta	*t*	*p*	*R* ^2^	Adj. *R*^2^	Conclusion
1	Emotional resonance	Sensible scene	0.45	0.049	0.48	9.12	<0.001	0.35	0.34	H4 supported
2	Cognitive evaluation	Imaginable atmosphere	0.43	0.050	0.44	8.56	<0.001	0.33	0.32	H5 supported
3	Behavioral intention	Virtual-real imagination	0.40	0.051	0.41	7.89	<0.001	0.31	0.30	H6 supported
Controls	All models	Gender; education level	-	-	n.s.	-	>0.05	-	-	Controlled

B, unstandardized coefficient; SE, standard error; *β*, standardized coefficient; *p*, significance probability. Gender and education level were entered simultaneously as control variables.

Adjusted *R*^2^ values ranged from 0.27 to 0.31 for the affordance-imagery models and from 0.30 to 0.34 for the imagery-response models. The sensible scene-emotional resonance association had the largest standardized coefficient (*β* = 0.48), whereas the perceived distributed affordance-virtual-real imagination association was smaller (*β* = 0.39). The latter pattern may be consistent with respondents’ lower familiarity with distributed authentication and digital derivative mechanisms. These comparisons concern coefficient magnitude within the present sample and should not be interpreted as evidence that one construct causally drives another.

Gender and education were not statistically significant in the focal models. This result means only that their partial coefficients were not distinguishable from zero under the present specification. It does not establish universality, rule out subgroup heterogeneity, or remove bias associated with voluntary online recruitment. The subsequent interaction analysis therefore focuses on technology acceptance as a pre-specified conditional correlate while retaining the same cautious interpretation.

### 4.4 Interaction and constrained serial indirect-association analyses

Hierarchical regression was used to examine whether the association between virtual-real imagination and behavioral intention varied with technology acceptance. Model 1 included gender, education level, virtual-real imagination, and technology acceptance (adjusted *R*^2^ = 0.35; *F* = 45.21, *p* < 0.001). Model 2 added the mean-centered interaction term, increasing adjusted *R*^2^ to 0.37 (Δ*R*^2^ = 0.02, *p* < 0.05). The interaction coefficient was *β* = 0.15 (*t* = 2.31, *p* < 0.05), indicating a stronger conditional association at higher reported technology acceptance. This is an attitude-based interaction in cross-sectional survey data, not evidence that actual blockchain or NFT exposure caused behavioral change.

**Figure 8:**
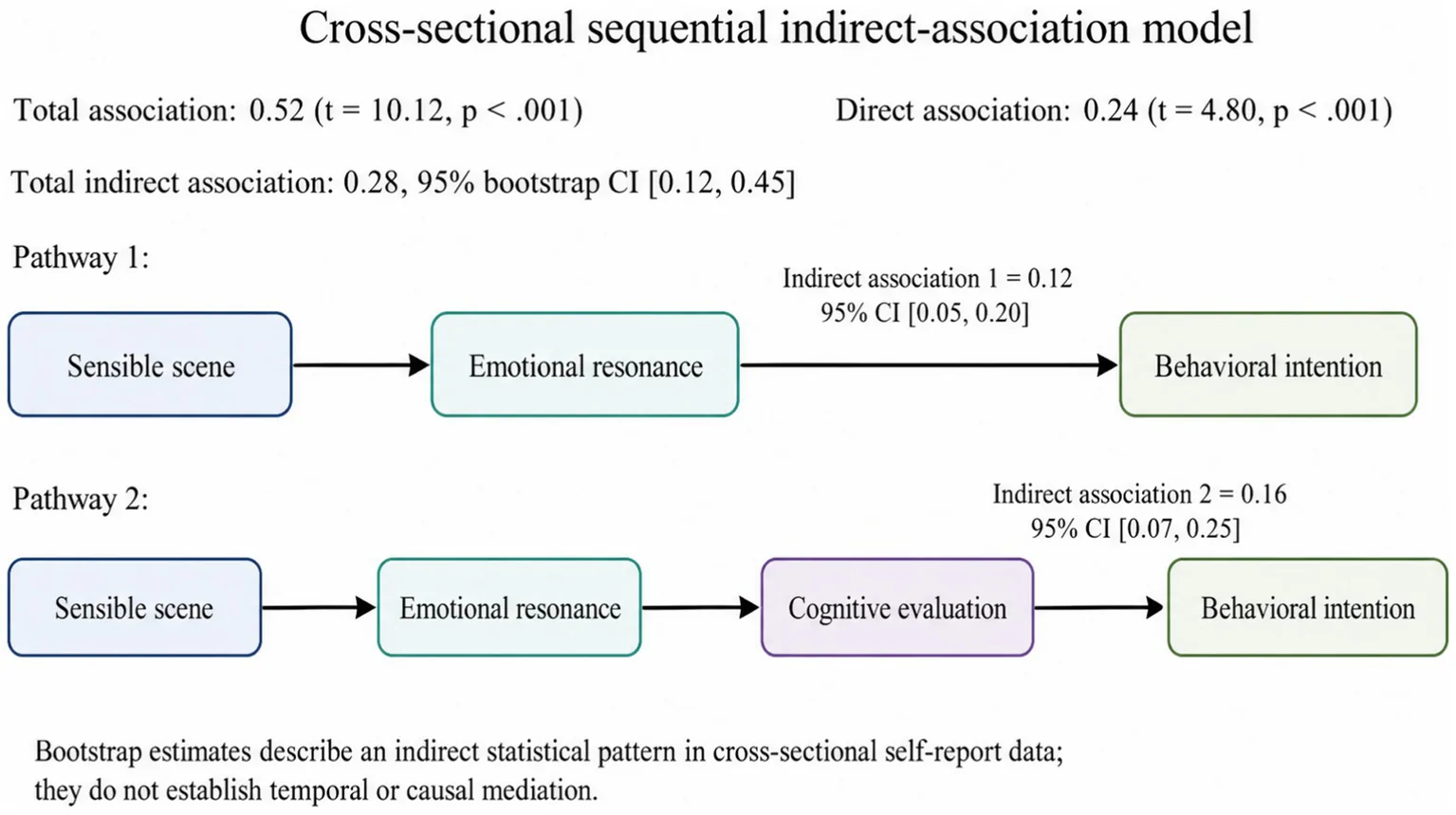
Constrained cross-sectional serial indirect-association model.

A prespecified constrained serial path model was estimated with 5,000 bootstrap samples to evaluate H7. The model included sensible scene → emotional resonance, emotional resonance → cognitive evaluation, emotional resonance → behavioral intention, cognitive evaluation → behavioral intention, and a direct sensible scene → behavioral intention path. A separate sensible scene → cognitive evaluation path was not estimated because the *a priori* theory positioned cognitive evaluation downstream of emotional resonance. Consequently, the two reported indirect components exhaust the indirect association in this constrained specification: sensible scene → emotional resonance → behavioral intention (0.12, 95% bootstrap CI [0.05, 0.20]) and sensible scene → emotional resonance → cognitive evaluation → behavioral intention (0.16, 95% bootstrap CI [0.07, 0.25]). The total association was 0.52 (*t* = 10.12, *p* < 0.001), the direct association was 0.24 (*t* = 4.80, *p* < 0.001), and the total indirect association was 0.28 (95% bootstrap CI [0.12, 0.45]) (Table 8; Figure 8). This decomposition identifies a statistically ordered cross-sectional pattern and does not establish temporal precedence or causal mediation.

**Table 8:** Bootstrap estimates for the constrained serial indirect-association model (*N* = 425).

Association component	Statistical path	Coefficient	SE	95% bootstrap CI	*p*
Total association	Sensible scene → behavioral intention	0.52	0.05	[0.42, 0.62]	<0.001
Direct association	Sensible scene → behavioral intention | indirect paths included	0.24	0.05	[0.14, 0.34]	<0.001
Indirect component 1	Sensible scene → emotional resonance → behavioral intention	0.12	0.03	[0.05, 0.20]	<0.001
Serial indirect component	Sensible scene → emotional resonance → cognitive evaluation → behavioral intention	0.16	0.04	[0.07, 0.25]	<0.001
Total indirect association	Sum of the two estimated indirect components	0.28	0.05	[0.12, 0.45]	<0.001

Bootstrap samples = 5,000. The constrained model did not estimate a separate sensible scene → cognitive evaluation path; therefore, the two reported indirect components sum to the total indirect association. A confidence interval excluding zero indicates statistical distinguishability from zero, not causal mediation.

## 5 Discussion and conclusion

### 5.1 Theoretical contribution

The seven hypotheses were evaluated within a survey-based media-psychology framework. H1-H3 were supported by positive adjusted associations between perceived immersive, interactive, and distributed affordances and sensible scene, imaginable atmosphere, and virtual-real imagination, respectively. H4-H6 were supported by positive associations between imagery characteristics and emotional resonance, cognitive evaluation, and behavioral intention. H7 was supported by a statistically distinguishable serial indirect component through emotional resonance and cognitive evaluation. The constrained decomposition also identified a shorter emotional-resonance-only component. These findings concern participants’ perceptions and expectations and do not demonstrate effects produced by actual VR, AR, NFT, blockchain, eye-tracking, or physiological interventions.

The theoretical contribution is an organizing account of how digitally framed affordances, artwork-related imagery perceptions, and audience-response indicators may be statistically connected. Compared with design-oriented discussions centered on platform functions, the TIP model separates perceived system capability from artwork quality and respondent response. The model therefore improves conceptual alignment with media psychology. However, it should be treated as a cross-sectional explanatory framework to be tested longitudinally or experimentally, not as a confirmed causal sequence.

### 5.2 Practical implications

The practical implications should be read as audience-informed design priorities rather than evidence of technological effectiveness. Respondents who reported stronger perceived immersive affordance also reported stronger sensible scene, suggesting that museums could prioritize testable prototypes involving controllable viewpoints, close-up access, and clear presentation of material layers. The association between perceived interactive affordance and imaginable atmosphere suggests that narrative exploration and participatory interpretation are appropriate candidates for user testing. Likewise, the association involving distributed affordance suggests that provenance, authorized sharing, and derivative participation may warrant transparent explanation. Each proposal requires evaluation with actual interfaces and behavioral outcomes before implementation claims can be made.

For educators, the results suggest testing lesson designs that pair perceptual attention with contextual interpretation, because emotional resonance and cognitive evaluation appeared in the observed indirect-association pattern. For technology developers, the findings identify expectations that may guide prototype development, but they do not show that VR simulations, parametric graphics, gesture recognition, or other devices improve learning or aesthetic response. Controlled comparisons should assess usability, accessibility, cybersickness, interpretive accuracy, and learning outcomes. Thus, the survey can inform what audiences expect from digital lacquer-painting experiences, whereas the effectiveness of any concrete technology remains an empirical question.

### 5.3 Research limitations and future directions

Several limitations constrain interpretation. First, all focal constructs were collected in a single cross-sectional self-report survey; temporal order, observed behavior, and causal mediation cannot be established, and common-method bias may remain despite the reported diagnostics. Second, the recruitment design was quota-guided but non-probability-based. Participants volunteered through art institutions, digital-art communities, and targeted interest advertising, creating self-selection and channel-selection bias. The sample was also highly educated and disproportionately interested in art or digital media. Consequently, the estimates are most applicable to engaged online audiences and should not be generalized to the wider public, infrequent technology users, or low-art-interest groups without replication. Third, the wording revisions and discriminant-validity tests reduce but cannot fully eliminate conceptual proximity among perceived affordance, imagery perception, and response constructs.

Future research should use probability-based or population-benchmarked sampling where feasible and should report nonresponse and weighting procedures. Experimental studies can compare actual VR, AR, web-based, and physical exhibition conditions, while longitudinal designs can test temporal ordering. Multimodal indicators such as fixation duration, pupil change, skin conductance, heart-rate variability, and interaction logs can complement self-report measures. Cross-cultural and low-technology-acceptance samples are also needed. These designs would allow the associations observed here to be evaluated as potential causal mechanisms rather than assumed to be such.

## Data Availability

The original contributions presented in the study are included in the article/Supplementary material, further inquiries can be directed to the corresponding author.
