# Supplementary material for: Audience attitudes and aesthetic perception of digitally empowered contemporary lacquer painting: a survey-based technology-imagery-perception model
Source: Front Psychol. 2026 Sep 3;17:1866076. doi: 10.3389/fpsyg.2026.1866076 (PMC13584868; doi:10.3389/fpsyg.2026.1866076)
Supplement: Supplementary file 1 [file Supplementary_file_1.docx]

Appendix A. Final Questionnaire Items

Reconstructed from the authors’ proof — author verification required before submission

# Response format and scope

**Response scale:** Seven-point Likert agreement scale (1 = strongly disagree; 7 = strongly agree). **Instrument structure:** 10 latent variables, each measured by three items (30 formal scale items in total). Technology-affordance items assess perceived system capabilities rather than prior use of a specific platform.

# Questionnaire items

| **Code** | **Construct** | **Item wording** |
| --- | --- | --- |
| **IA1** | Immersive affordance | A digital interface could let me zoom, rotate, or switch viewpoints when examining a lacquer painting. |
| **IA2** | Immersive affordance | A digital display system could let me inspect the luster, texture, and layered details of a lacquer painting from controllable viewing distances. |
| **IA3** | Immersive affordance | A digital viewing environment could provide a sense of mediated presence while I examine a lacquer painting. |
| **IN1** | Interactive affordance | A digital platform could let me select different information paths when exploring a lacquer painting. |
| **IN2** | Interactive affordance | A digital interface could let me compare different details, interpretations, or versions of a lacquer painting. |
| **IN3** | Interactive affordance | A digital platform could enable viewers to contribute interpretations or responses. |
| **DA1** | Distributed affordance | Digital authentication tools could provide traceable provenance records for a lacquer painting. |
| **DA2** | Distributed affordance | A digital platform could record licensing and authorized-use information for a lacquer painting. |
| **DA3** | Distributed affordance | A networked system could provide authorized access to verified digital representations of a lacquer painting. |
| **SS1** | Sensible scene | The lacquer painting appears to have tangible material texture. |
| **SS2** | Sensible scene | The layered surfaces and luster of the lacquer painting are visually perceptible. |
| **SS3** | Sensible scene | The lacquer painting conveys visual depth and spatial concreteness. |
| **AT1** | Art-temporal scene | The lacquer painting evokes a clear narrative setting. |
| **AT2** | Art-temporal scene | The lacquer painting encourages associations with its cultural context. |
| **AT3** | Art-temporal scene | The lacquer painting creates a meaningful situational atmosphere. |
| **VR1** | Virtual-real scene | The physical lacquer painting and its digital representation feel conceptually connected. |
| **VR2** | Virtual-real scene | The material and digital versions of the lacquer painting appear to belong to a coherent whole. |
| **VR3** | Virtual-real scene | The lacquer painting maintains a recognizable identity when it circulates between physical and digital forms. |
| **ER1** | Emotional resonance | The lacquer painting evokes a strong emotional response in me. |
| **ER2** | Emotional resonance | I feel emotionally engaged when viewing the lacquer painting. |
| **ER3** | Emotional resonance | The lacquer painting resonates with me personally. |
| **CE1** | Cultural empathy | The lacquer painting helps me understand its cultural meaning. |
| **CE2** | Cultural empathy | I can interpret the ideas expressed by the lacquer painting clearly. |
| **CE3** | Cultural empathy | Viewing the lacquer painting encourages me to reflect on its artistic and cultural value. |
| **BI1** | Behavioral intention | I would be willing to share information about this lacquer painting. |
| **BI2** | Behavioral intention | I would be willing to revisit this lacquer painting or related presentations. |
| **BI3** | Behavioral intention | I would recommend this lacquer painting or participate in related activities. |
| **TA1** | Technology acceptance | I am familiar with digital technologies used to present or communicate artworks. |
| **TA2** | Technology acceptance | I believe digital technologies are useful for understanding and engaging with lacquer painting. |
| **TA3** | Technology acceptance | I believe digital technologies for viewing lacquer painting would be easy to use. |

# Construct abbreviations

IA = Immersive affordance; IN = Interactive affordance; DA = Distributed affordance; SS = Sensible scene; AT = Art-temporal scene; VR = Virtual-real scene; ER = Emotional resonance; CE = Cultural empathy; BI = Behavioral intention; TA = Technology acceptance.
